# Photodynamic therapy mediates antitumor effects through multiple non‑apoptotic cell death pathways

**DOI:** 10.1186/s12929-026-01222-5

**Published:** 2026-07-15

**Authors:** Kaili Liao, Xiaofei Lin, Hui Liang, Yi Lin, Shuai Liu, Xiwen Yan, Xinrui Liu, Minqi Zhu, Jingyi Gan, Xiya Yan, Zijia Li, Yanxin Luo, Xiaozhong Wang, Jiasheng Xu

**Affiliations:** 1 Jiangxi Province Key Laboratory of Immunology and Inflammation, Jiangxi Provincial Clinical Research Center for Laboratory Medicine, Department of Clinical Laboratory, The Second Affiliated Hospital, Jiangxi Medical College, Nanchang University, Nanchang, Jiangxi China; 2 School of Stomatology, Nanchang University, Xuefu Road, Nanchang, 330006 Jiangxi China; 3 The First Clinical Medical College of Nanchang University, Xuefu Road, Nanchang, 330006 Jiangxi China; 4 The Second Clinical Medical College, Nanchang University, Nanchang, 330006 Jiangxi China; 5 Queen Mary College of Nanchang University, Xuefu Road, Nanchang, 330006 Jiangxi China; 6 School of Public, Health of Nanchang University, Xuefu Road, Nanchang, 330006 Jiangxi China; 7 Department of Colorectal Surgery, The Second Hospital of Zhejiang University School of Medicine (Key Laboratory of Cancer Prevention and Intervention, China National Ministry of Education; and Key Laboratory of Medical Molecular Biology, Zhejiang Province), Zhejiang University, Hangzhou, 310009 China; 8 The Fourth Clinical Medical College of Nanchang University, Xuefu Road, Nanchang, 330006 Jiangxi China

**Keywords:** Photodynamic therapy (PDT), Non-apoptotic cell death, Ferroptosis, Pyroptosis, Reactive oxygen species (ROS), Hypoxia-responsive agents

## Abstract

Photodynamic therapy (PDT) demonstrates remarkable versatility by activating diverse non-apoptotic cell death pathways, effectively circumventing apoptosis resistance and enhancing tumor eradication. Key mechanisms include autophagic cell death, regulated necrosis-driven immune activation, ferroptosis-mediated oxygen replenishment, pyroptosis-induced immunogenic cell death (ICD), and paraptosis. These pathways collectively highlight PDT’s capacity to disrupt tumor survival mechanisms and stimulate systemic anti-tumor immunity. However, several challenges remain, including precise spatiotemporal control of ROS, hypoxia mitigation, and selective photosensitizer delivery. Advances in nanotechnology, hypoxia-responsive agents, and combination therapies (e.g., immune checkpoint inhibitors or chemotherapy) hold promise for overcoming these limitations. In this review, we discuss multiple types of non-apoptotic cell death pathways activated by PDT and the underlying mechanisms of each distinct cell death process. We also review the clinical applications and current challenges of leveraging these pathways to enhance tumor treatment in PDT. Future research should prioritize the development of subcellular-targeted photosensitizers, deeper light-penetration technologies, and biomarker-guided personalized regimens for more precise and effective PDT.

**Graphical abstract:**

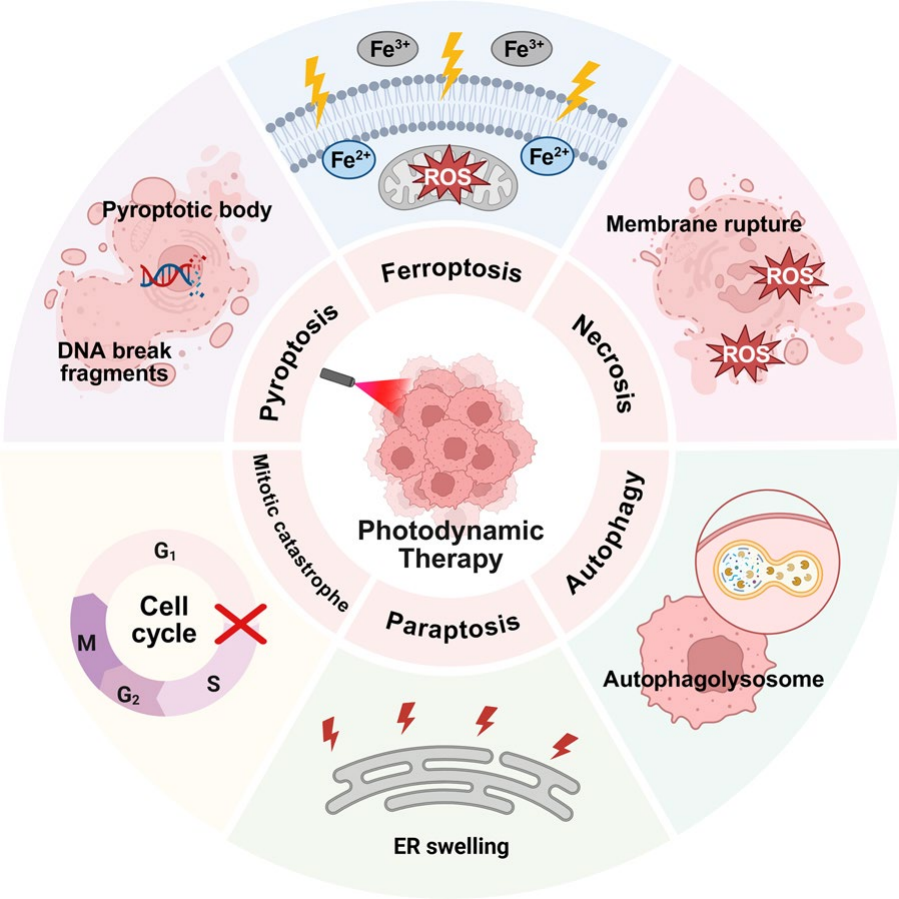

## Introduction

Photodynamic therapy (PDT), as a strategy for tumor treatment, has garnered significant interest due to its advantages of minimal invasiveness, targeting and controllability. Its core mechanism relies on the generation of Reactive Oxygen Species (ROS) by photosensitizers under the activation of light at a wavelength corresponding to an absorbance optimum of the photosensitizer, which directly kills tumor cells or destroys the tumor microenvironment through oxidative damage [1]. PDT has evolved into a sophisticated technology and is now widely used in clinical practice for the treatment of malignant including gastrointestinal, skin, head and neck, and gynaecological cancers, non-malignant such as psoriasis, and pre-malignant lesions such as actinic keratoses [2–4]. Apoptosis has long been considered as the main pathway of PDT-induced tumor cell death. However, recent studies have shown that the resistance to apoptosis of tumor cells may be a potential limiting factor for the efficacy of PDT [5–7], and that multiple non-apoptotic cell death pathways induced by PDT also play a key role in the anti-tumor effect, and even dominate in some cases.

Non-apoptotic cell death is highly heterogeneous and includes necroptosis, autophagy-dependent death, ferroptosis, pyroptosis, ICD, etc. These pathways differ significantly in their molecular mechanisms, morphological features and functional effects, but can be activated by PDT-triggered signaling networks such as oxidative stress, mitochondrial damage or endoplasmic reticulum stress.

Among the various pathways of cell death induced by PDT, apoptosis predominates; however, the pathways of cell death in PDT are not singular but are collectively influenced and regulated by multiple factors [8, 9]. Firstly, the type of the photosensitizer directly determines the initial site of damage, thereby activating different death pathways. Secondly, the light dose affects the mode of cell death: high light doses generate large amounts of ROS, leading to rapid and severe damage that often results in necrosis, whereas low light doses produce fewer ROS, acting more gently and typically inducing apoptosis [10]. Finally, the cell type determines the sensitivity and response to PDT. For instance, some cancer cells may have defective apoptosis mechanisms, making them more prone to switch to necrosis or other death pathways [11].

Therefore, this review aims to comprehensively elucidate the diverse non-apoptotic cell death pathways of tumor cell under PDT, and discuss the detailed mechanisms of the damage of tumor cells caused by PDT, so as to provide valuable insights into the clinical application of PDT and promote the development of PDT in the field of tumor therapy.

## Autophagy

### Definition of autophagy

Autophagy is a process of degradation and recycle of damaged cellular components in eukaryotes, which is usually induced by various forms of cellular stress such as hypoxia, starvation, and metabolic disorders [12]. Autophagy is divided into three types: macroautophagy, microautophagy and chaperone-mediated autophagy [13]. Moderate autophagy functions as a self-repair mechanism, enhancing adaptability and viability under harsh environmental conditions. Conversely, excessive autophagy can result in cell demise, a process termed Autophagic Cell Death or Type II programmed cell death [14–16].

### Mechanism of autophagy induced by PDT

**Fig. 1:**
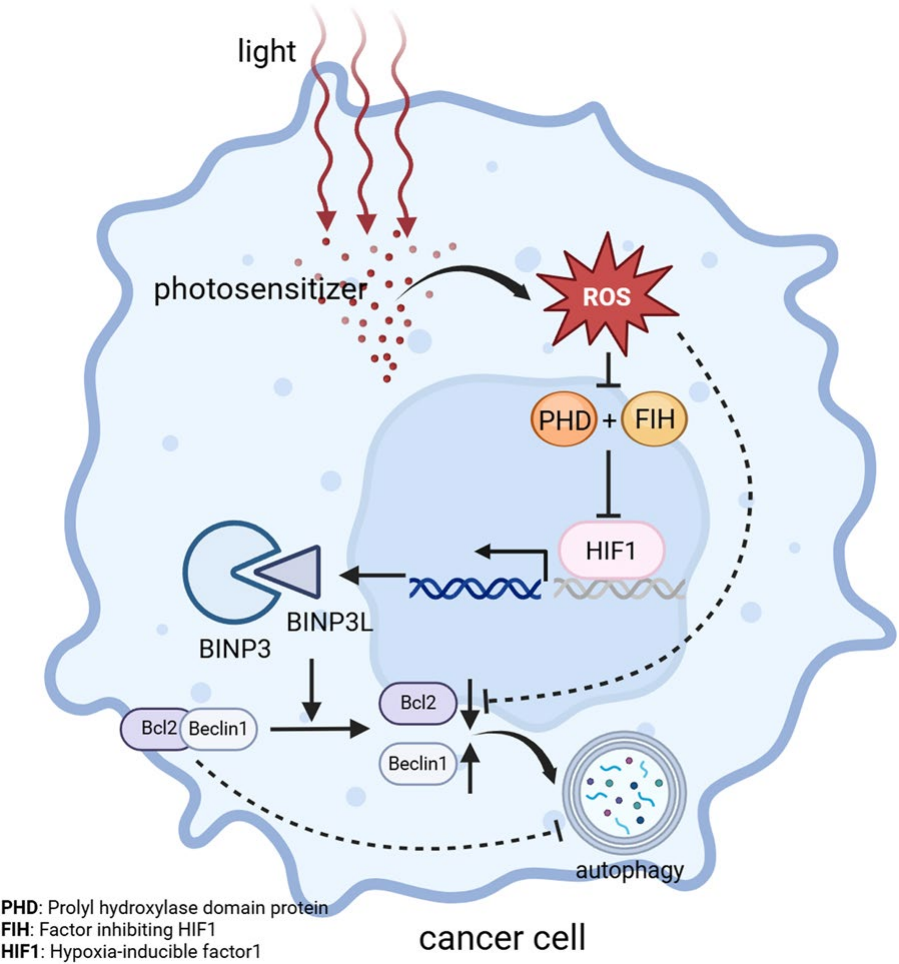
ROS-HIF1-BNIP3/NIX-mediated autophagy pathway in cancer cells during PDT. Under normoxic conditions, PHD and FIH inactivate HIF1 through hydroxylation. PDT-mediated activation: **1** Light activates photosensitizer, generating ROS and inducing intracellular hypoxia. **2** ROS inhibit enzymatic activities of PHD and FIH. **3** Accumulated HIF1 activates transcription of BNIP3 and BNIP3L. **4** Newly synthesized BNIP3/BNIP3L proteins competitively bind to Beclin1-Bcl2 complexes via atypical BH3 domains. **5** Released Beclin1 initiates autophagosome formation

Autophagy is involved in the process of PDT and plays an important role in PDT. Photosensitizers accumulate in the tumor cells and are activated by light of appropriate wavelength. Photosensitizers transfer electrons to oxygen molecules to generate ROS, such as singlet oxygen (^1^O_2_), superoxide anion (O_2_^·−^), hydroxyl radical (·OH), and hydrogen peroxide (H_2_O_2_). ROS is considered to be a key molecule in the activation of autophagy by PDT. The accumulation of ROS in cell will induce oxidative damage and mitochondrial dysfunction, which constitute a major stress sign to initiate autophagy [17, 18]. However, the specific molecular mechanism of its regulation of autophagy remains unclear. One possible pathway is ROS-HIF1-BNIP3/NIX-autophagy [19] (Fig. 1). Under normoxic conditions, PHD (Prolyl hydroxylase domain protein) and FIH (Factor inhibiting HIF1) inactivate HIF1 (Hypoxia-inducible factor 1) through hydroxylation. PDT induces intracellular hypoxia and generates ROS, which inhibit the activity of PHD and FIH, leading to an elevation in HIF1 levels. Activated HIF1 induces the transcription of BNIP3 and BNIP3L, whose atypical BH3 domains compete for binding with the Beclin1-Bcl2 and Beclin1-Bcl-XL complexes, thereby releasing Beclin1 and enhancing autophagy. The mechanism of PDT-induced autophagy also involves mammalian target of rapamycin (mTOR) [20]. The mTOR complex plays a pivotal role in regulating cell growth and proliferation and its pharmacological inhibition can induce autophagy. In PDT, the amphiphilic photosensitizer AlPcS(2a) directly targets the mTOR signaling network. Treatment with AlPcS(2a)-PDT results in a rapid and partial depletion of both total mTOR and its phosphorylated form at Ser^2448^ in both cultured cells and tumor xenografts. This attenuation of mTOR levels and phosphorylation significantly alleviates its inhibitory effect on autophagy, thus facilitating the induction of autophagy [21].

### The pro-survival effect of autophagy in PDT for tumors

#### Stress-adaptive response

PDT generates ROS via photosensitizer activation, inducing oxidative stress in tumor cells. In response to this stress, autophagy is activated [22, 23]. Moderate autophagy can enhance the viability of tumor cells, enabling them to survive in environments of oxidative stress, thus reducing the therapeutic effect of PDT. Studies have shown [22, 24] that autophagy enhances the stress adaptability of tumor cells during PDT through the following mechanisms: **(a)** Clearance of damaged organelles or macromolecules: Autophagy alleviates the damage of oxidative stress to cells by degrading mitochondria and protein aggregates impaired by ROS. **(b)** Maintenance of energy homeostasis: Autophagy recycles intracellular components to provide energy and metabolic substrates for cells, helping tumor cells survive under stress conditions induced by PDT. **(c)** Inhibition of apoptosis: Autophagy can inhibit the initiation of the apoptosis program in tumor cells through the recycling of photodamaged mitochondria before they can initiate apoptosis. If autophagy is inhibited, usually by a genetic alteration, this will inhibit the recycling process, resulting in a higher level of photokilling [20].

#### The development of drug resistance

In tumor treatment, the mechanism underlying PDT resistance has attracted considerable attention, and autophagy plays a crucial role in it [25]. PDT damages tumor cells by generating ROS, while simultaneously activating autophagy. The PDT-induced activation of autophagy can clear oxidized cellular components, leading to a reduction in tumor sensitivity to PDT, thereby compromising therapeutic efficacy [26]. Research by Kessel D et al. [27] showed that under low-dose PDT, the survival rate of Atg7-knockdown L1210 cells—a model with deficiency in the core autophagy gene Atg7—was significantly lower than that of wild type L1210 cells. In addition, in colorectal cancer stem-like cells, PDT can induce the formation of autophagosomes and the upregulation of autophagy-related proteins. Inhibiting autophagy can significantly promote apoptosis, revealing the cytoprotective effect of autophagy [28].

### The pro-death effect of autophagy in PDT

Although autophagy is typically regarded as a cytoprotective pathway, accumulating evidence demonstrates that excessive autophagy can conversely induce autophagic cell death. The hyperactivation of autophagy can lead to massive degradation of crucial cellular components, causing irreversible cell damage and death [29]. Buytaert et al. [30] revealed that in apoptosis-deficient cells a nonapoptotic pathway dependent on sustained autophagy commits the oxidatively damaged cells to death. Lopes et al. [31] found that after the treatment of renal cancer cells with berberine combined with PDT, a large number of ROS could be detected. The Autophagy Assay Kit analysis indicated the fluorescence intensity of autophagosomes significantly increased, and the survival rate of renal cancer cells significantly decreased, suggesting that autophagy can promote the killing effect of PDT. Excessive autophagy can contribute to cell death through the following potential mechanisms [32]: **(a)** Excessive ER-phagy-mediated cell death. Persistent degradation of endoplasmic reticulum fragments impairs ER function, leading to dysregulation of lipid and cholesterol metabolism [33]. **(b)** Mitophagy. Hyperactivation of mitophagy results in mitochondrial dysfunction and promotes cell death [34]. **(c)** Autosis. This form of autophagy-dependent cell death exhibits distinctive morphological features including elevated autophagic vacuolation and localized swelling in the perinuclear region. Its underlying mechanism is closely linked to the activity of Na⁺, K⁺-ATPase [35].

### Challenges and strategies in clinical applications

#### Precise regulation of the timing and degree of autophagy

ROS-induced autophagy during PDT exhibits a dual role in tumor treatment. On the one hand, moderate autophagy can protect tumor cells. On the other hand, excessive autophagy leads to cell death. Therefore, precisely regulating the timing and degree of autophagy is the key to improve the efficacy of PDT. Chen et al. [36] developed a nanomedicine (CeCe) by assembling a photosensitizer (Chlorin e6, Ce6) and an autophagy promoter (Celastrol). They demonstrated CeCe could generate abundant ROS and elevate the autophagy level to initiate autophagic cell death under light irradiation. More importantly, by employing autophagy inhibitors, the anti-proliferation ability of CeCe decreased, suggesting that the cell death induced by CeCe was autophagy-dependent, thereby proposing a synergistic antitumor strategy of PDT and autophagy promotion. Besides, photo-responsive nanomaterials can be activated by light of appropriate wavelength to precisely regulate the spatiotemporal distribution of autophagy. For instance, certain nanomaterials can generate local thermal effects or ROS under light irradiation, thus inducing the autophagic death of tumor cells while reducing damage to normal tissues [37]. Moreover, combining PDT with chemotherapy and immunotherapy can further optimize autophagy regulation.

#### Personalized treatment

Tumor diseases are highly heterogeneous. The characteristics of tumor cells, the microenvironment, and the responses to treatment vary significantly among different patients. Therefore, personalized treatment is crucial for improving the efficacy of tumor treatment [23]. The tumor microenvironment has a significant impact on the efficacy of PDT. Hypoxic environments can affect autophagy and confer resistance to PDT in tumors. Specifically, hypoxia stimulates pro-survival autophagy through pathways involving the activation of HIF1 and HIF2α [38], which helps tumor cells mitigate metabolic stress and thus survive the cytotoxic conditions. Changes in the tumor microenvironment can indirectly influence the efficacy of PDT by affecting inflammatory and immune response [39]. When combining PDT with autophagy regulation, drugs that improve hypoxia can be used, or photosensitizers and autophagy regulators insensitive to hypoxia can be selected. He et al. found that in the acidic tumor microenvironment, a bovine serum albumin and platinum compound can release platinum and exerts a catalytic activity that produces oxygen, thereby alleviating hypoxia and augmenting the efficacy of PDT [40]. Shi et al. developed a histone deacetylase-targeted photosensitizer capable of overcoming hypoxia and intracellular oxidative resistance [41]. Personalized treatment requires a comprehensive assessment of the tumor’s attributes, including anatomical depth, histopathology, and microenvironmental conditions, etc. For example, in the treatment of head-and-neck tumors and skin tumors, the selection of appropriate photosensitizers, administration methods, or combination with autophagy regulators should be tailored based on anatomical location, disease stage, histopathological type, and photosensitivity [42].

## Necrosis

### Definition of cell necrosis

Cell necrosis is a form of non-programmed cell death characterized by the loss of plasma membrane integrity, leading to the leakage of cellular contents, including proteases, thereby triggering a local inflammatory response that is avoided by apoptosis [43, 44]. While apoptosis is a primary and programmed cell death pathway triggered by moderate-does PDT, particularly through the release of cytochrome c from damaged mitochondria [45], cell necrosis typically occurs at higher PDT doses through cell membrane destruction and lysis [46]. Its main morphological features include cell swelling, organelles destruction, and concomitant cytoplasmic leakage [44].

Necroptosis shares morphological features with necrosis, such as plasma membrane rupture and leakage of cellular contents, yet fundamental distinctions exist between these processes. Necrosis typically results from pathological acute tissue damage and triggers robust inflammatory responses. In contrast, necroptosis represents a programmed form of cell death that serves as a crucial defense mechanism against specific pathogen invasions. The canonical necroptosis pathway is mediated by death receptors involving the RIPK1-RIPK3-MLKL axis, activated through specific signaling pathways and governed by precise genetic programming. Furthermore, necroptosis demonstrates significant relevance to cancer pathogenesis, and its dysregulation may contribute to various human diseases, including cancers [47–49].

### Molecular mechanism of PDT-induced cell necrosis

Photosensitizers are activated under light and generate abundant ROS. ROS can oxidize lipids, proteins and DNA in the cell. Lipid peroxidation disrupts membrane integrity and function, leading to a loss of cellular homeostasis. When this damage becomes overwhelming, the cell is unable to maintain its normal physiological processes, culminating in necrotic cell death [50–52]. ROS can not only directly damage cell structures, but also regulate cell survival and death by activating specific signaling pathways, such as NO pathway, Akt/mTOR and cAMP/ protein kinase A signaling pathways [51, 53]. Studies have shown that PDT-induced necrosis can also trigger an acute inflammatory response, promote leukocyte infiltration at the tumor site, and enhance antigen presentation, thereby further activating the systemic anti-tumor immune response [50, 54–56].

### Regulation of cell necrosis in PDT

Necrosis was initially considered to be an unregulated type of cell death. However, growing evidence now indicates that it can be a regulated process and not just a catastrophic event. The most studied regulated necrosis is necroptosis, which involves specific signaling pathways and molecular mechanisms that can be targeted to control cell death. Receptor-interacting protein 1 (RIP1) and receptor-interacting protein 3 (RIP3) are two critical regulators of necroptosis [57]. In PDT, ROS promotes the formation of necrosomes and induces necroptosis by regulating the phosphorylation and oxidation of RIP1 and RIP3 [53]. It has been found that granular nucleus formation is inhibited by specific necrosis inhibitors such as necrostatin-1, suggesting that this nuclear morphology is part of a regulated necrosis process [58]. In PDT, the amount and range of ROS generation and action can be precisely controlled by adjusting the type and dose of photosensitizer and light conditions, thereby optimizing the balance between cell necrosis and survival. Since excessive ROS can cause damage to normal tissues, precise control is required to maximize antitumor effects and minimize side effects.

### Existing results of cell necrosis induced by PDT

#### Subcellular localization of photosensitizers and necrosis

Studies have shown that the subcellular localization of photosensitizers is closely related to the cell death mode. Soriano et al. [58] investigated the photodynamic effect of zinc phthalocyanine (ZnPc) in HeLa cells in vitro. They found that subtle differences in incubation times can result in different intracellular localization and then different cell death processes. Short-term PS incubation induces necrosis when ZnPc is near the plasma membrane during uptake and internalization, whereas prolonged treatment results in necroptosis when ZnPc accumulated in the Golgi. Valli F et al. [59] investigated the mechanism of action of zinc (II) phthalocyanine photosensitizers (Pc13) in PDT. They found that ROS produced by Pc13 under light can induce apoptosis and necrosis in melanoma cells. It has been shown that the intracellular localization of Pc13, especially its distribution in mitochondria and lysosomes, significantly affects the efficacy of PDT. Kim et al. [52] developed membrane fusogenic liposomes (MFLs) to selectively deliver photosensitize ZnPc to the plasma membrane of cancer cells. The ZnPc-loaded MFLs showed higher phototoxicity compared to conventional liposomes (i.e., non-fusogenic cationic liposomes) that deliver ZnPc to intracellular compartments via endocytosis. The membrane localization of ZnPc resulted in the local generation of a large number of ROS, significantly damaging the plasma membrane and causing cell necrosis. This study suggests the potential application of membrane-localized photosensitizers in the treatment of cancer.

#### PDT-induced necrosis in various cancers

Several studies have shown that PDT is effective in inducing cell necrosis across various types of cancer cells. Leviskas B et al. [60] demonstrated that Pd(T4), a palladium-based metalloporphyrin photosensitizer, was effective in inducing necrosis and apoptosis of highly aggressive uveal melanoma cells (C918) when used alone or in combination with 5-ALA under 405 nm blue light irradiation. This provides new ideas for the potential application of PDT in combating highly aggressive cancers. Gao et al. [50, 61] used chlorophyll derivatives (5, 10, 15, 20-tetraphenyl-[2:3]-[(methoxycarbonyl, carboxy) methano] chlorin I and 5, 10, 15, 20-tetraphenyl-[2:3]-{[methoxycarbonyl, (2 hydroxyethyl) amide] methano} chlorin II) in PDT. They demonstrated that they can effectively induce the necrosis of cholangiocarcinoma cells under 650 nm light, especially compound I exhibited significant anti-tumor activity, showing the potential clinical application value of this method. Marydasan B et al. [62, 63] used chlorophyll derivative 5, 10, 15, 20-tetrakis (3, 4-dihydroxyphenyl) chlorin (TDC) in PDT to validate that it can effectively induce the necrosis of ovarian cancer cells under near-infrared light, which further demonstrated its potential in clinical application. Huggett et al. successfully induced necrosis in locally advanced pancreatic cancer by PDT using Verteporfin. Studies have shown that Verteporfin has good safety and feasibility, short drug-light interval and less skin photosensitivity, proving its application prospect in the treatment of advanced pancreatic cancer [64]. Er O et al. successfully induced apoptosis and necrosis of pancreatic cancer cells using ZnPc-loaded mesoporous silica nanoparticles for PDT, proving the great promise of PDT in the treatment of metastatic cancer [65].

#### Combination strategies

Photosensitizer combined therapy strategy can significantly improve the anti-tumor efficacy by combining different treatment methods to synergistically enhance the killing effect of PDT on tumor cells. Acedo et al. [66] achieved significant cellular photoinactivation in three tumor cell lines (HeLa, HaCaT, and MCF-7) by PDT using a combination of zinc (II)-phthalocyanine (ZnPc, which primarily localizes to the Golgi apparatus) and cationic porphyrin (TMPyP, which primarily localizes to lysosomes) as photosensitizers. The two photosensitizers could both be irradiated using a light source of the same wavelength (λ = 650 nm). This combination treatment induced either apoptosis or necrosis, depending on the light dose. The results indicated that the strategy of combining two photosensitizers significantly improved the efficacy of PDT, demonstrating its potential as an innovative cancer treatment. Popescu et al. [67] studied the therapeutic effect of PDT in combination with the COX-2 inhibitor Parecoxib. They found this combination significantly increased necrosis and apoptosis indices in tumor tissue and enhanced the therapeutic effect by increasing oxidative stress and reducing antioxidant defenses. Compared with PDT alone, the combined treatment group showed stronger anti-tumor activity, holding promise to optimize clinical treatment regimens.

In conclusion, the necrosis mechanism induced by PDT can cause loss of cell membrane integrity and mitochondrial dysfunction through oxidative damage to critical organelles such as tumor cell membrane and mitochondria by ROS, which can trigger programmed necrosis and directly destroy tumor cells. This mechanism can further activate the host immune response by destroying tumor blood vessels and triggering a local acute inflammatory response. Therefore, PDT-induced necrosis shows unique advantages in the treatment of cancers, especially those that are resistant to other treatments. Furthermore, the irradiation wavelength is critical for effectively inducting necrosis. Besides matching the absorbance optimum of the photosensitizer, the wavelength of light directly determines the tissue penetration depth. Longer wavelengths penetrate tissue more deeply than shorter wavelengths. For instance, red light penetrates skin more deeply than blue light, but its photon energy is lower [46]. Therefore, an appropriate light wavelength needs to be selected to optimize the therapeutic effect. Future research should focus on the optimization and distribution of photosensitizers, maximize the therapeutic effect of necrosis by precisely controlling the dose and light conditions of PDT, and explore the combination of PDT with other treatment methods to further improve its clinical effect.

## Ferroptosis

### Definition of ferroptosis

Ferroptosis is a new form of cell death. In 2003, Dolma et al. discovered a small molecule drug, Erastin, that could induce tumor cell death [68]. The morphology of this drug-induced cell death is distinct from those of other known forms of cell death: it is characterized by mitochondrial atrophy and an increase in lipid reactive oxygen species. Moreover, this death process cannot be blocked by inhibitors of apoptosis, necrosis and autophagy [69]. In 2012, Professor Brent Stockwell called this new form of cell death “Ferroptosis” [70].

Disruption of intracellular iron metabolism and the accumulation of lipid peroxidation (LPO) are the main causes of ferroptosis [71]. Under normal circumstances, the amount of iron ions in and outside the cell remains relatively stable, which is known as iron homeostasis. When the supply of iron exceeds the demand of the cell, the iron homeostasis is imbalanced. Excess iron will produce a large amount of ROS under the action of the Fenton reaction. ROS will further react with biological membranes to produce LPO, which will damage biological macromolecules such as DNA and proteins, causing ferroptosis [72].

The glutamate-cysteine transport system transports cysteine into the cell, where it is reduced to cysteine and used to synthesize glutathione. Glutathione, as an endogenous antioxidant, assists glutathione peroxidase 4 (GPX4) in removing LPO accumulated in the cell, thereby achieving the effect of anti-oxidation and protecting cells [71]. When cells undergo ferroptosis, various causes lead to a decrease in GSH, and excess LPO cannot be cleared, resulting in cell death [73].

Taking advantage of the characteristics of ferroptosis, inducing ferroptosis in cancer cells through various pathways can be used to fight tumors. At present, a variety of drugs, such as sorafenib, artemisinin, sulfasalazine and nano-iron oxide particles, have been discovered and used to induce ferroptosis in cancer cells [74–78], and have played a therapeutic role in various cancers.

### The synergistic effect of ferroptosis and PDT in anti-tumor

#### Similarities between the mechanisms of ferroptosis and PDT

In PDT, the photosensitizers are activated by light and then generate cytotoxic ROS, which cause the oxidation and destruction of biomolecules in cancer cells, thereby killing them [79]. Ferroptosis is an iron-dependent form of regulated cell death driven by LPO. Upon the induction of ferroptosis, cellular iron homeostasis is disrupted, leading to a significant release of free iron, primarily through sources such as ferritinophagy and the hyperactivation of transferrin receptor 1 (TfR1). The accumulated Fe^2^⁺ catalyzes the Fenton reaction with intracellular hydrogen peroxide (H_2_O_2_): Fe^2^⁺ + H_2_O_2_ → Fe^3^⁺ + ·OH + OH⁻. The generated ROS can directly attack intracellular polyunsaturated fatty acids (PUFAs), thereby initiating lipid peroxidation [80, 81]. Additionally, when GPX4 activity is lost or intracellular GSH is depleted, LPO accumulate uncontrollably, ultimately disrupting membrane integrity and causing ferroptosis. Both ferroptosis and PDT cause the accumulation of ROS in cancer cells, which leads to oxidative damage to biomacromolecules and damage to cell membranes, thereby killing cancer cells or suppressing the proliferative activity and invasiveness of cancer. Studies have shown that the two have a synergistic effect in fighting tumors [82]. PDT can disrupt cell redox homeostasis not only by generating cytotoxic ROS, but also depleting intracellular GSH to induce ferroptosis. The synergistic antitumor effects arise from PDT’s direct cytotoxic action, while ferroptosis constitutes an indirect secondary mechanism.

#### PDT-induced ferroptosis

PDT can induce cell death through a variety of cell death pathways, including ferroptosis [83, 84]. Researchers have found that in triple-negative breast cancer cells after PDT treatment, there is a depletion of GPX4 and peroxidation of membrane lipids, which are characteristic of ferroptosis [85]. It has been showed that the ferroptosis inhibitor deferiprone can significantly block PDT-induced cell death, indicating that PDT can induce ferroptosis [86].

On the one hand, when PDT is used to treat tumors, the photosensitizer can undergo two photochemical reactions to produce ROS under laser irradiation. Type I photochemical reactions excite the photosensitizer and transfer electrons or protons to form hydrogen peroxide; in type II photochemical reactions, the photosensitizer is excited to the triplet state, where it interacts with molecular oxygen to produce singlet oxygen [87, 88]. Both hydrogen peroxide and singlet oxygen can directly or indirectly induce ferroptosis. The former can maintain the Fenton reaction during ferroptosis, while the latter can directly oxidize the phospholipids on the cell membrane, causing LPO accumulation and inducing ferroptosis [89].

On the other hand, PDT can downregulate the expression of SLC7A11 and GPX4 in cells [90]. Some prior studies revealed that PDT treatment upregulates *p53*, which subsequently suppresses SLC7A11 expression at the transcriptional level, thereby inhibiting cellular cystine uptake [91]. Additionally, research has shown that PDT induces robust lymphocyte infiltration within tumors and stimulates interferon-γ (IFN-γ) secretion. This cytokine further downregulates membrane-localized SLC7A11, enhancing tumor cell susceptibility to ferroptosis. Reduced cystine uptake impairs GSH biosynthesis [92]. Since GPX4 expression and activity are directly governed by GSH availability, GSH depletion triggers GPX4 inactivation, resulting in uncontrolled lipid peroxidation and ROS accumulation, thereby inducing ferroptosis [93]; the System Xc-/GSH/GPX4 may play a crucial role in PDT-induced ferroptosis [89].Other researchers have found that genes regulating PDT can control the ferroptosis process. By investigating RNA sequencing data from cholangiocarcinoma cells after PDT treatment, the researchers found that PDT can upregulate the expression of ferroptosis-related genes SLC2A1, SLC2A6, and SLC7A5, and inhibit the expression of ZEB1, inducing ferroptosis [94]. The application of certain photosensitizers alone on tumors can also induce their ferroptosis. 5-ALA can induce ferroptosis in cancer cells by downregulating GPX4 and upregulating heme oxygenase-1 (HMOX1). Similarly, verteporfin has been shown to promote ferroptosis through GPX4 downregulation. As photosensitizers, they can also induce ferroptosis in the absence of light [95, 96]. However, different cancer cells have different sensitivities to ferroptosis.

The accumulation of ROS in PDT is necessary for the induction of ferroptosis. However, the process is regulated by many other factors. The susceptibility to ferroptosis of cells, the choice of photosensitizer and parameters of PDT can all affect the induction of ferroptosis [82, 97]. In short, PDT can induce ferroptosis in cancer cells, but the complex mechanism of action has not yet been fully elucidated. Many studies have shown that the combination of PDT with ferroptosis-promoting therapy is more effective than PDT alone, providing new insights into improving anti-tumor outcomes [98–100].

### Ferroptosis compensates for the shortcomings of PDT

#### Limitations of PDT

In the actual clinical application of PDT, its limitations are reflected in many aspects. For example, tumor sites are often hypoxic, and the lack of oxygen causes insufficient generation of ROS by PDT [101, 102]; the low bioaccumulation of traditional photosensitizers (such as hematoporphyrin) results in insufficient effective concentrations in tumors, which limits the effect of PDT and also causes toxic side effects; and the absorption of light by tissues interferes with the conversion of light energy by photosensitizers [103]. All of these factors reduce the efficacy of PDT in tumor treatment.

#### Ferroptosis relieves tumor hypoxia

The molecular biological characteristics of ferroptosis can be used to compensate for the deficiencies and inadequacies of PDT. On the one hand, in cells undergoing ferroptosis, the synthesis of GSH is blocked, and LPO disrupts the redox balance of the cell, which jointly enhances the efficacy of PDT [104]. On the other hand, during ferroptosis, iron ions flow inward, and the excessive iron ions in the cell react with hydrogen peroxide in the tumor cells through the Fenton reaction to produce ROS and oxygen, which can provide oxygen for PDT, relieve the hypoxic environment at the tumor site, and further enhance the efficacy of PDT [105].

At present, there have been many studies on ferroptosis to compensate for the shortcomings of PDT. These studies combine ferroptosis inducers such as erastin, sorafenib with PDT photosensitizers, prepare them into nanoparticles and apply them to tumor treatment, achieving good results [106, 107]. Another study embedded photosensitizer Ce6 and hemin into the AS1411 G-quadruplex (G4) unit to form a DNA nanozyme. This nanozyme mimics catalase activity by catalyzing the decomposition of intracellular H_2_O_2_ into oxygen, thereby alleviating hypoxia, which was confirmed by the suppression of hypoxia-inducible factor-1α. Simultaneously, it induces ferroptosis through GSH depletion and LPO accumulation, ultimately achieving synergistic antitumor efficacy [108].

#### Application of combined ferroptosis and PDT in tumor treatment

Recently, a variety of drugs that combine ferroptosis and PDT have been developed, most of which are nanoparticles, including polymers, metal organic frameworks, inorganics, carrier-free NPs, and biomimetic materials (nanoscale particles designed by mimicking the structure, composition, or function of organisms) [109]. For example, T. Zhu et al. assembled the photosensitizer Ce6 with the ferroptosis inducer Erastin to form a nanomedicine, combining the induction of ferroptosis in tumor cells with PDT effectively inhibits the activity of tumor cells [110]. In order to further alleviate the hypoxic state at the tumor site, some researchers have combined haemoglobin with Ce6 and used it in combination with PDT to treat tumors. In the constructed nanoplatform, hemoglobin was pre-saturated with oxygen, enabling it to carry oxygen, which can then be released at the tumor site to alleviate the hypoxic state. Simultaneously, hemoglobin can serve as an iron source to induce ferroptosis in tumor cells [92]. Another researcher designed a novel hypoxia-responsive nanoreactor, BCFe@SRF, which covalently links the photosensitizer Ce6 with bovine serum albumin and ferritin via azobenzene and is loaded with the ferroptosis inducer sorafenib to provide better therapeutic effects for synergistic PDT and ferroptosis therapy [111]. In the hypoxic tumor microenvironment, the nanoreactor is activated and synchronously releases Ce6, Ferritin, and Sorafenib. This process directly generates ROS through the photodynamic effect, while simultaneously utilizing iron-catalyzed Fenton reactions and SRF to inhibit the cellular antioxidant defense, collectively exacerbating lipid peroxidation and thereby creating synergistically amplified oxidative damage. The experimental results show that the combination therapy group exhibited the strongest cancer cell-killing effect in vitro and achieved the most significant tumor growth inhibition in tumor-bearing mice, fully demonstrating the synergistic effect between PDT and ferroptosis.

## Pyroptosis

### Definition of pyroptosis

Pyroptosis is a form of programmed cell death driven by inflammation. The typical pathway is mediated by NLRP3 inflammasome: the intracellular sensor NLRP3 senses signals such as metabolic abnormalities/mitochondrial damage, recruits and activates caspase-1, which cleaves gasdermin-D (GSDMD) to form a pore, and in concert with IL-1β triggers cell lysis and inflammatory factor release. The atypical pathway is triggered by the direct cleavage of GSDMD by caspase-4/5 leading to cell lysis and release of inflammatory mediators, and can also reverse activate NLRP3 inflammasome. Both pathways exacerbate the inflammatory response by regulating IL-1β/IL-18 secretion and LDH release [112].

### Pathways of PDT-induced cellular pyroptosis

**Fig. 2:**
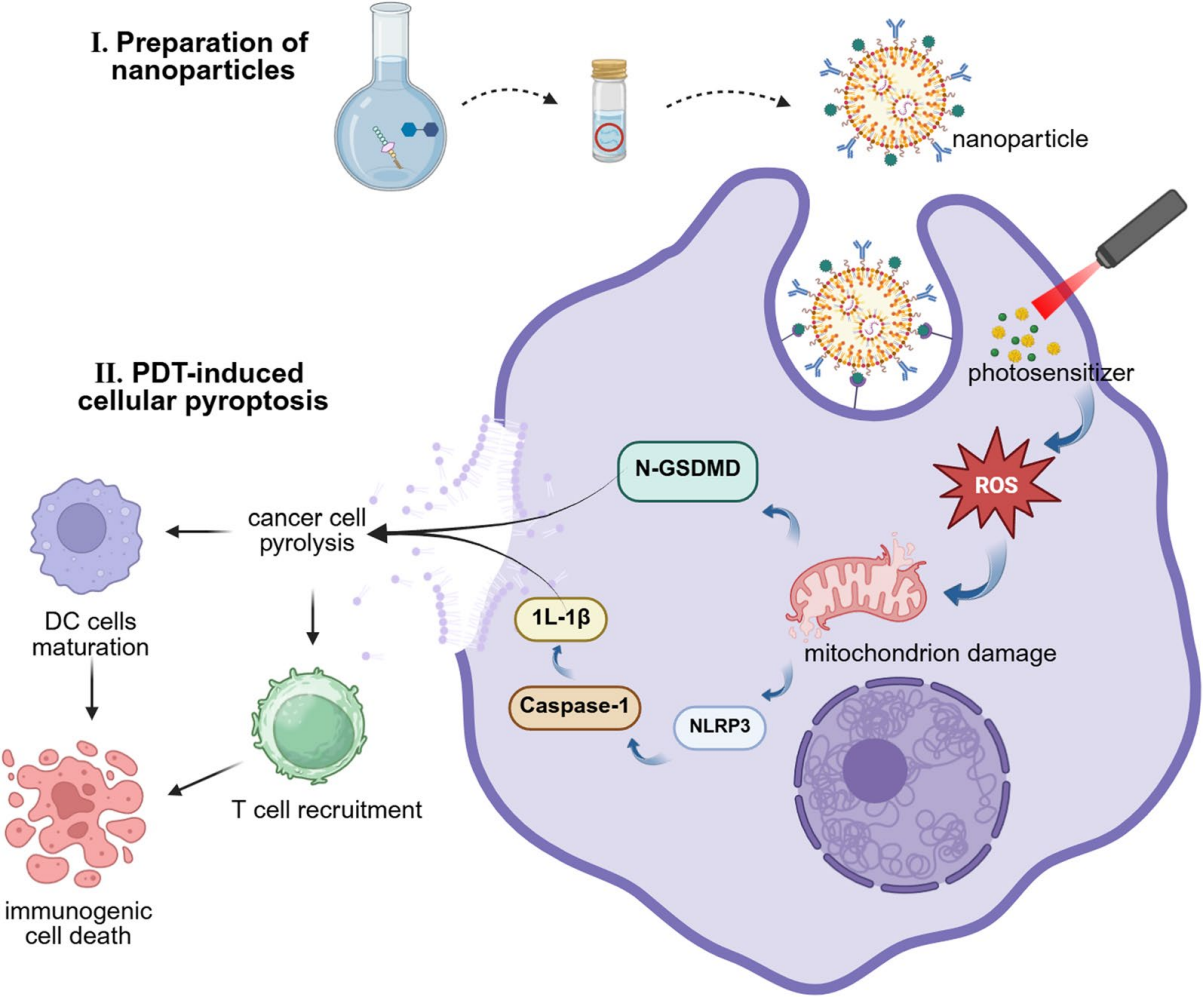
Light-activated nanoplatform for ROS-triggered pyroptosis and immunotherapy via mitochondrial damage and NLRP3 inflammasome activation. **I**: through reasonable design, the researchers combined drugs and photosensitizers to build a nano-platform. **II**: under the irradiation of laser, photosensitizer stimulates the generation of a large amount of ROS to trigger pyroptosis, and at the same time achieves the controllable release of the drug. Intracellular ROS overload leads to severe mitochondrial damage and increase of mitochondrial ROS. The oxidative damage of mitochondria triggers NLRP3, and NLRP3 further activates Caspase-1 to cut Pro-1L-1β to form 1L-1β, and cleave GSDMD to release N-GSDMD. N-GSDMD binds to the cell membrane to form perforation, which, together with 1L-1β, drives cell rupture and cell factor release. The released cytokines further stimulate DC cell maturation, T cell recruitment and immunogenic death

PDT induced cellular pyroptosis by the pathway: ROS-NLRP3-GASPASE-1-GSDMD, which has been described above. PDT resulted in intracellular ROS overload and severe mitochondrial damage as evidenced by a decrease in MMP, an increase in mitochondrial ROS, and the release of mitochondrial DNA (mtDNA) into the cytosol. The release of mtDNA activates inflammatory vesicles (NLRP3), and then NLRP3 activates caspase-1. Caspase-1 activation cleaves proIL-18 and NLRP3 activation in the cytosol. Activation cleaves proIL-18 and proIL-1β and ultimately promotes their maturation [113]. Activation of inflammatory cysteine proteases also cleaves GSDMD and releases the N-terminal gasdermin-D (N-GSDMD), which forms holes in the cell membrane through perforation upon binding to the cell membrane, leading to cell swelling, membrane rupture, release of cellular contents (e.g., LDH, IL-1β, IL-18), and cell death [114].In turn, the released DAMPs and inflammatory factors further induce DC (dendritic cell) maturation and T-cell recruitment, thereby inducing immunogenic death (Fig. 2).

### Advantages of PDT-induced cellular pyroptosis over apoptosis

In contrast to apoptotic cell shrinkage, pyroptosis is hallmarked by pronounced cellular swelling [115]. PDT usually induces apoptosis. Immunogenicity is poor due to slow or even no release of DAMPs [116].

Most photosensitizers must be used in combination with Immune Checkpoint Blockade (ICB) to achieve satisfactory anti-tumor immune effects compared to their use alone. The lack of tumor antigens leads to low response rates, which is the main challenge of ICB therapy. In recent years, ICB therapies have demonstrated good efficacy in various cancers. Unfortunately, immunocold tumors respond poorly to ICB therapy. Research methods to improve the response of immunocold tumors to ICB therapy are a major focus in oncology research [117]. Pyroptosis has pro-inflammatory effects and can trigger strong anti-tumor immunity. Pyroptosis has received much attention as a form of ICD. In ICB, cold tumors (tumors resistant to immune checkpoint inhibitors) can be transformed into reactive hot tumors in the accompaniment of pyroptosis, suggesting that pyroptosis-induced inflammation increases the efficiency of ICB and effectively inhibits tumor progression. Due to factors such as the complexity of the immune microenvironment, the combination of PDT photosensitizers and ICB is not commonly used in clinical practice, but it is still considered a promising cancer treatment strategy.

### Pathways to achieve targeted induction of cellular pyroptosis

Cellular pyroptosis, an inflammatory and catalytic form of cell death, is an effective means of anti-tumor therapy; however, cellular pyroptosis is usually activated by chemotherapeutic agents, limiting its anti-tumor application due to resistance and severe side effects. Due to the ubiquitous expression of gasdermin-E (GSDME) throughout the body, untargeted triggering of pyroptosis induces systemic side effects and toxicity. The heterogeneity of the tumor microenvironment is the main reason for ineffective tumor therapy and uncontrolled tumor progression. ICD therapy based on pyrolysis is an ideal strategy to overcome tumor microenvironment heterogeneity and achieve satisfactory anti-tumor effects. However, the efficiency of current pyroptosis-based therapies, which mainly rely on a single endogenous or exogenous stimulus, is limited by the intrinsic pathological characteristics of malignant cells. Therefore, there is a need to develop a synergistic strategy with high tumor specificity and modulability.

#### Finding potential targets

Starting from the mechanism of PDT-induced tumor cell pyroptosis, we search for potential targets for treating tumors, bringing new protocols and strategies for treating tumors. Based on the evidence of retinal pigment epithelial cell damage in age-related macular degeneration (AMD) in vitro, preclinical models, and clinical studies of AMD, Yang Ming et al. hypothesised that novel programmed cell deaths including pyroptosis could be a new target for AMD treatment [118].And by exploring the mechanism of ALA-PDT for anti-acne in vitro, Zhang Jian and other scholars identified the therapeutic target as the follicular sebaceous gland unit, which catalyses the generation of ROS, which leads to the assembly of NLRP3 inflammasomes and the activation of caspase-1, and identified PDT-induced cellular pyroptosis as a potential new approach for anti-acne in vitro. However, the mechanism of PDT-induced cellular pyroptosis at the molecular level is still unclear, and studies based on this could provide valuable ideas for PDT treatment of different tumors [119]. Chen Diyan et al. found that Modified 5-aminolevulinic acid photodynamic therapy (M-PDT) induced apoptosis in cutaneous squamous cell carcinoma (cSCC), and M-PDT promoted pyroptosis by up-regulating the NLRP 3/GSDMD pathway, confirming that c-Jun N-terminal kinase (JNK) is a promising target for M-PDT-induced pyroptosis, which provides a new pathway and theoretical basis for improving the efficacy of PDT [119]. By exploring the specific mechanism of GSDME-mediated apoptosis in PDT of esophageal squamous carcinoma, Lisa Li et al. demonstrated that PDT can down-regulate the expression of PKM 2, which activates caspase-8 and caspase-3, and ultimately releases N-GSDME and triggers apoptosis in esophageal squamous cell carcinoma (ESCC) through in vivo and in vitro experiments [120].

#### Development of new photosensitizers

Conventional photosensitizers tend to result in a single programmed cell death process, leading to insufficient photodamage, which severely hampers their application. The development of novel photosensitizer drugs, or the modification of traditional photosensitizers, mediates the generation of ROS by tumor cells, targeting the activation of cellular pyroptosis mechanisms and reducing tumor cell survival. By observing the effects of Tetra-α-(4-carboxyphenoxy) phthalocyanine zinc (TαPcZn)-mediated PDT (TαPcZn-PDT) on cytotoxicity, cell viability, apoptosis, intracellular ROS, mitochondrial membrane potential, caspase-1, caspase-3, and nuclear transcription factor-κB (NFκB) in MCF-7 cells, Chunjie Ma et al. found that TαPcZn-PDT resulted in increased cytotoxicity, cell apoptosis and cell pyroptosis, decreased cell viability and Δ Ψm, and ROS generation as well as activation of caspase-1, caspase-3, and NFκB. And siRNA targeting of caspase-1 (siRNA-caspase-1) attenuated the effects of TαPcZn-PDT mentioned above [121]. IR 700DX-6T, a photosensitizer that targets mitochondrial translocation proteins, may trigger colorectal cancer antitumor immune response-induced pyroptosis. IR 700DX-6T induces pyroptosis by promoting the phosphorylation of downstream p38 and the cleavage of gasdermin E (GSDME) mediated by active caspase 3 (CASP3). IR 700DX-6T combined PDT enhances the sensitivity of MSS-1000 colorectal cancer cells to PD-L1 blockers, can inhibit the development of colorectal cancer in vitro and in vivo, and provides a good idea for the treatment of colorectal cancer [122].

However, the photosensitizer still lacks targeting in activating cellular pyroptosis, based on which the researchers designed the photosensitizer to achieve its localisation to subcellular structures. To achieve photo-activated cancer cell pyroptosis, Min Wu et al. developed three membrane anchoring photosensitizers with aggregation-induced emission characteristics (AIE PSs), which could emit bright fluorescence with effective ROS generation in aggregate states and were denoted TBD-R, with different numbers of cationic chains. The TBD-R photosensitizers were synthesized by conjugating 1,1,2,2-tetraphenylethene-benzo[c] [1,2,5]thiadiazole-2-(diphenyl methylene) malononitrile (TPE-BT-DC, TBD) and phenyl rings with cationic chains, where the TBDs supported the dual properties of fluorescence turn-on imaging and photosensitisation to PDT, and the cationic chains enabled the probe to be inserted into the cell membrane. With the increase in the number of cationic chains, the membrane anchoring ability increased. In vitro experiments demonstrate that TBD-3C (TBD decorated with three cationic groups) has a strong membrane-anchoring ability, which enables the in situ generation of ROS, directly damaging the membrane and thereby leading to cancer cell death [123]. Wang Hong and other scholars found that LDH@ZnPc, obtained through isolation of ZnPc using positive charged layered double hydroxides (LDH), can be used as an excellent apoptosis inducer with mitochondrial anchoring ability, which can cause mitochondrial oxidative stress and thus enhance ICD for enhanced PDT and enhanced anti-tumor immunity through both in vitro and in vivo settings [124]. Zeng Shuang and other scholars have designed the smart molecule ER-ZS for endoplasmic reticulum targeting diagnosis and therapy in cellular and animal models. ER-ZS is a type I photosensitizer that produces O_2_⋅- and ⋅OH upon light irradiation, which is obtained by combining hemicyanine dyes with ER-targeted functional groups (p-toluenesulfonamide). Thus, after a certain time of irradiation, PDT causes severe oxidative damage to the ER of tumor cells in hypoxic (2% O_2_) conditions and activates a unique pathway of pyroptosis that showed excellent anti-tumor ability in xenograft tumor models, providing new ideas for molecular optical integration of non-alcoholic fatty liver disease (NAFLD) diagnosis and cancer therapy [125]. In addition, Chuang et al. used molecular engineering of electron donors to develop a plasma membrane and mitochondria dual-targeted NIR-II photosensitizer (DPITQ), with quinolinium as the fixed acceptor while 3,3-dimethyl-N,N-diphenyl-3H-indol-5-amine substituted thiophene as the donors, for dual-targeted hypoxic tumor therapy at the plasma membrane and mitochondria by stimulating synergistic pyroptosis and apoptosis. Good biocompatibility and appropriate lipophilicity help DPITQ to specifically anchor to the plasma membrane and mitochondria of cancer cells in complex biological scenarios. DPITQ treated with 635 nm laser can disrupt the intact plasma membrane in both cell experiments and in mice, leading to mitochondrial dysfunction and ultimately to concomitant pyroptosis and apoptosis, thereby inhibiting cancer cell proliferation, even under hypoxic conditions [126]. The above researchers have enhanced the targeting of photosensitizer-induced cellular pyroptosis in the treatment of tumors by achieving the localization of the photosensitizer to different subcellular structures.

Nonetheless, the above photosensitizers still could not overcome the obstacles that prevented the widespread use of PDT for tumor therapy, such as the inability of some photosensitizers to differentiate between tumors and healthy tissues, and the limited depth of tissue penetration of the light source, so Tan Yubo et al. designed and introduced a two-photon photosensitizer (TPSS), which specifically targets tumors overexpressing carbonic anhydrase IX, thus showing a particular specificity for tumor cells. Its excellent photosensitivity at low photon density and two-photon absorption properties. The simultaneous absorption of two photons leads to enhanced treatment depth and increased effectiveness of PDT. Upon exposure to 480 nm single-photon and 915 nm two-photon laser irradiation, TPSS can generate a large amount of ROS in vitro and in vivo, induce cell pyroptosis, and subsequently trigger a strong anti-tumor immune response. This strategy overcomes the limitations of PDT and increases treatment depth and effectiveness [127]. However, the application of two-photon system in PDT is limited because it depends on irradiation equipment which is difficult to popularize.

#### Building a collaborative nano platform

Nanotechnology-based pyroptosis shows great advantages in this regard, in terms of enhanced therapeutic efficacy and improved patient response rates.

(1) Controlled drug release at the tumor lesion through rational design to avoid systemic toxicity.

The non-targeted distribution of traditional chemotherapy drugs and photosensitizers can damage healthy tissues, leading to systemic toxicity. When PDT induces massive destruction of tumor cells, the released harmful substances and cellular contents may also trigger systemic inflammatory responses and immune reactions. Developing nanoscale platforms enables controlled drug release and targeted delivery of photosensitizers, thereby reducing damage to healthy tissues and systemic toxicity. Conventional photosensitizers with highly conjugated plane structure are hindered by the quenching effect in the aggregated state, which significantly reduces the fluorescence and the efficacy of PDT. Liu Dongfang et al. designed and constructed a hypoxia-responsive covalent organic frameworks (COF) platform, which quenched the fluorescence of aggregation-induced emission luminogen (AIEgen) loaded into nanopores and reduced the efficacy of PDT via the photoinduced electron transfer (PET) effect. AIEgen actually is a series of donor–acceptor (D–A) type molecules based on triphenylamine (TPA) with varying numbers of electron-withdrawing substitutes, specifically, one, two, and three 2-(thiophen-2-ylmethylene)malononitrile (TM) groups. Hypoxia triggered the degradation of azocarbonyl COF, which resulted in a significant enhancement of both the fluorescence and PDT properties of AIEgens, accompanied by the controlled release of decitabine (DAC) drug. DAC can promote tumor cell thermal apoptosis induction by up-regulating the GSDME expression to promote induction of thermal apoptosis in tumor cells. Therefore, the integration of DAC and AIEgen-based PDT could effectively amplify the induction of pyroptosis in tumor cells, achieving the activation of luminescence with therapeutic effect and the release of self-accelerating drug, thus advancing synergistic cancer immunotherapy [128]. Yao Xiao and other scholars designed a novel tumor microenvironmental ROS/GSH dual-responsive nanoplatform (MCPP) to release anti-tumor immune responses by inducing pyroptosis to improve the efficiency of immune gateway blockade and achieve control of tumor growth. The MCPP loaded with cytotoxic agent paclitaxel and phototoxic agent purpurin 18 had good ability to induce pyroptosis. In addition, the ROS generated by purpurin 18 after laser irradiation achieved controlled drug release [129]. Jiang Xiaoyan and other researchers prepared Bi_2_Te_3_ nanoparticles with good stability by hydrothermal reaction (180 °C temperature), with K_2_TeO_3_ and BiCl_3_ as raw materials. Due to the superior photothermally-driven thermoelectrocatalytic properties of Bi_2_Te_3_ nanoparticles, they showed a highly effective tumor synergistic therapeutic effect based on photothermal therapy (a main approach utilized for phototherapy using photosensitizers to destroy tumor cells without damage to normal tissues by the heat generated through photothermal conversion) and large amount of ROS generation under laser irradiation. Controlled drug release was achieved by constructing a nanoplatform to avoid systemic toxicity and mitigate the side effect of PDT-induced pyroptosis [130].

(2) Tumor metastasis is an inevitable problem, and nanotechnology may enhance anti-tumor immunity in vivo, especially after laser irradiation, which induces oxidative stress in tumor cells and reverses the immunosuppressive state of tumors. Enhancement of nanotechnology-based pyroptosis has received increasing attention in terms of tumor immunogenicity and induction of systemic immunity. PolyMN-TO-8, a supramolecular nanomicelle assembled by Wang Xing and others using methoxypolyethylene glycol 2000, the anti-inflammatory prodrug, can induce immunogenic cell death mediated by photothermal decomposition under laser irradiation while avoiding the occurrence of immune dysregulation. This therapeutic approach activates pro-inflammatory factors to an appropriate extent, eliminates immune escape, enhances dendritic cell maturation, and accelerates the infiltration of CD8+ T cells into tumors. Evaluation of anti-tumor activity in mice showed that the aforementioned therapy, through synergistic effects of PDT, photothermal therapy, and chemotherapy, achieved a tumor inhibition rate as high as 87.44% [131]. Yang Jianquan et al. constructed albumin nanospheres (named IR 780-ZnS@HSA) with a photosensitizer-IR 780 encapsulated in the nucleus and a cGAS-STING agonist/H2S generator-ZnS loaded on the shell. In vitro, IR 780-ZnS@HSA generates therapeutic effects through photothermal therapy and PDT. In addition, it stimulates ICD and activates pyroptosis in tumor cells via the caspase-3-GSDME signalling pathway.IR 780-ZnS@HSA also activates the cGAS-STING signalling pathway. These two pathways synergistically enhance the immune response [132]. In addition, Qiu Wei et al. constructed MRC nanoparticles, pH-responsive nanoparticles loaded with the immunomodulator RGX-104 and the classic photosensitizer Ce6. These nanoparticles are induced by acidic tumor microenvironment (TME), can exhibit faster drug release efficiency at pH 5.0 (simulating mature lysosomes) compared to pH 6.8 (simulating primary endosomes) and pH 7.4 (simulating normal physiological conditions), combining controlled drug release with enhanced anti-tumor immunity. RGX-104 not only eliminates myeloid-derived suppressor cells via the liver-X nuclear hormone receptors/ApoE pathway, but also enhances macrophage phagocytosis and dendritic cell (DC) maturation by releasing small molecules like ATP. This process reshapes the tumor microenvironment (TME), thereby creating conditions that exacerbate pyroptosis. Ce6-triggered PDT enhances oxidative stress and organelle destruction to increase immunogenicity. In vivo and in vitro experiments have confirmed that the immunomodulatory photodynamic MRC nanoparticles will implement the aforementioned dual strategy to enhance gasdermin E-dependent pyroptosis [133].

(3) The inability of visible light to penetrate biological tissue limits its use in phototherapy and photodiagnosis of deep tissue sites of disease. There have been reports demonstrating the beneficial effects of light-emitting diode (LED) irradiation in various pathologies, including cancer [134]. A potential solution is to use miniaturized implanted LEDs to bring the light source closer to the disease site, but it is currently unclear whether implantable wireless LEDs offer any clinical improvements over fine optical fibers that can also be introduced into deep tissue locations. Sunghoon Rho has fabricated an LED-based wireless device that has the ability to excite a green absorbing dye, Bengal rose (type II photosensitizer). By using wireless devices as light sources and attaching them to the back of the cell culture medium, with Bengal rose as the photosensitizer, irradiating for 30 min at room temperature can induce death of the cultured human colon adenocarcinoma cell line HT-29 cells. The researchers found that prominent bubbles were observed over time in the light-activated cells, indicating cell death by light-induced pyroptosis, and supporting evidence was obtained by staining the cells with Annexin-V, FITC and propidium iodide fluorescent probes. This result reveals a way forward for an LED-based wireless implantable device that triggers photodynamic ICD in deep cancer tissue [135]. The use of micro-implantable LEDs to bring light sources closer to the diseased site is expected to overcome the limitations of limited tissue penetration of light, while targeted implantation can also achieve precise local treatment. However, implantable light sources require surgical insertion and removal, and ensuring the stability and reliability of the device in the body remains a major challenge.

## Mitotic catastrophe

Mitotic catastrophe has been widely used to describe cell death due to aberrant mitosis [136], and there are currently three main mechanisms: first, mitotic disorder leads to the initiation of apoptotic mechanisms [137]. Second, mitotic catastrophe triggers a lethal pathway. In this case the cell will die in the interphase into the next cell cycle [138]. Third, abnormal mitosis initiates the cellular senescence programme [139–141]. In some articles it has been suggested that damage to DNA and proteins required in mitosis (e.g., spindle, microtubule proteins, etc.) may have a pathway to mitotic catastrophe [142]. PDT, because of its ability to produce ROS to damage DNA and proteins, has been suggested to be used for inducing mitotic catastrophe as a low-resistance antitumor therapy.

Some studies have shown that PDT may induce mitotic catastrophe in cells through several mechanisms: PDT causes abnormal chromosome segregation by generating large amounts of ROS, which can damage microtubules and spindle inside the cell; the ROS generated can also directly damage DNA molecules, thus triggering mitotic catastrophe [143]. In some studies, it has also been found that PDT may arrest the cell cycle during G2/M, the mechanism of which may be the phosphorylation of WEE1 after DNA damage, which inhibits the interaction of WEE1 with SIRT1, a protein kinase important for cell cycle checkpoints, and the aberrant phosphorylation of this enzyme prevents the cell from completing normal mitosis, causing cell death [144, 145].

## Paraptosis

Although Sperandio’s research team discovered a new form of cell death distinct from traditional apoptosis in 293 T cell lines overexpressing insulin-like growth factor receptor in 2000 [146, 147], it was not named “paraptosis” in the updated cell death nomenclature system by the NCCD in 2018 due to the lack of effective biomarkers. Despite this, as a form of programmed cell death, it still holds ample research space and application potential. In recent years, utilizing PDT to induce paraptosis in tumor cells has become a research direction for relevant researchers.

### Mechanism of paraptosis

Paraptosis is a non-classical form of programmed cell death, characterized primarily by the swelling and vacuolization of the endoplasmic reticulum and mitochondria. Unlike apoptosis, paraptosis does not involve the activation of caspases, chromatin condensation, or cellular fragmentation. In contrast to autophagy, the vacuoles formed during paraptosis are surrounded by a single membrane layer, rather than a double membrane layer.

Paraptosis can be triggered by multiple factors, with the following four being the primary ones:

Reduced intracellular protein degradation: This leads to the accumulation of misfolded proteins in the endoplasmic reticulum lumen, causing cellular stress overload and resulting in ER swelling and vacuolization, ultimately leading to cell death [148].

Imbalance in thiol redox homeostasis: The formation of native disulfide bonds is a rate-limiting step in the proper folding of many proteins. An imbalance in thiol redox homeostasis increases the occurrence of protein misfolding, leading to the accumulation of misfolded proteins and subsequent ER stress-induced cell death.

Mitochondrial calcium overload: This causes changes in mitochondrial osmotic pressure, leading to swelling and even vacuolization of the mitochondria. The number and size of these vacuoles are time-dependent, ultimately resulting in cell death.

Excessive generation of ROS: This can also induce paraptosis in cells [149].

Based on existing research, paraptosis is highly associated with protein synthesis and can be blocked by the translation inhibitor cycloheximide [150]. The process is also accompanied by changes in calcium ion levels and redox homeostasis. Most paraptosis phenomena depend on the mitogen-activated protein kinase (MAPK) family, including c-Jun N-terminal kinase 1 (JNK1), p38, and mitogen-activated protein kinase 2, which can be inhibited by the multifunctional adaptor protein Alp-1/Alix [151, 152]. Additionally, recent studies on PDT have found that paraptosis following endoplasmic reticulum photodamage is caused by both MAPK-dependent and MAPK-independent pathways [153].

### Paraptosis and PDT

In PDT-mediated paraptosis of tumor cells, it primarily involves the induction of single-membrane-bound vacuoles, leading to cell death. Since the formation of vacuoles depends on gene transcription and translation processes, blocking the corresponding pathways can inhibit the paraptosis process [154]. Therefore, relevant studies have shown that when using low-dose verteporfin-PDT, MAPK inhibitors and protein synthesis inhibitors can inhibit the formation of single-membrane vacuoles. However, it is worth noting that the signaling pathways of paraptosis are mostly independent of these factors in clinical settings [147, 155]. Additionally, in strict PDT treatment methods that result in cell death rates higher than LD90, endoplasmic reticulum proteins undergo cross-linking, leading to reduced protein mobility in the endoplasmic reticulum, thereby inhibiting the formation of typical single-membrane vacuoles and suppressing the paraptosis pathway [153, 156].

In the localization of photosensitizers in PDT-mediated paraptosis, it is generally speculated that photosensitizers mainly target the endoplasmic reticulum due to their association with misfolded proteins [157]. Multiple studies have confirmed this hypothesis. For example, after treatment with ER-targeted photosensitizers such as HPPH, hupericin, and verteporfin, followed by PDT using LD90 dose of m-tetrahydroxyphenylchlorin (m-THPC), paraptosis was observed in human non-small cell lung cancer A549 cells and mouse liver cancer cells [158, 159]. However, there are also studies indicating that photosensitizers located in other cellular compartments can induce paraptosis. For instance, in PDT using chlorin NPE6, which is localized to lysosomes, paraptosis was not observed, further supporting the above speculation [155]. Interestingly, in PDT using [Ru(bipy)2-dppz-7-methoxy][PF6]2 (Ru65), paraptosis characteristics were observed, even though the photosensitizer was localized to the nucleus [160].

With the increasing research on PDT therapy and cell death in recent years, significant progress has been made in the study of PDT and paraptosis. In the latest research, it has been found that thiol oxidants (such as GSH) can reduce the rate of paraptosis or even block it. Researchers speculate that reducing high GSH expression in cells can promote paraptosis and contribute to its role as an anti-tumor therapy [161]. In addressing the inhibitory effect of tumor hypoxia on the effectiveness of PDT, researchers have developed a nano-platform called GC@MCS NPs, which consists of hypoxia-responsive hyaluronic acid-nitroimidazole (HA-NI), MnO_2_ NPs as an oxygen regulator, and poly (L-glutamic acid) derivatives (γ-PFGA) as the core delivery system for gambogic acid (GA) and Ce6. They found that PDT using this nano-platform effectively generate ROS and deeply penetrates cancer cells, leading to the death of 4T1 tumor cells both in vitro and in vivo [82, 146, 162]. In this process, there is an accumulation and fusion of vacuoles in the endoplasmic reticulum and mitochondria, indicating paraptosis.

Furthermore, elevated ROS levels induced by PDT reduce intracellular GSH levels, and the decrease in GSH levels further leads to the accumulation of ROS, which can also trigger other types of cell death, such as ferroptosis, by affecting the GSH-redox system in response to GC@MCS NPs-based PDT [163]. However, a decrease in GSH levels may lead to adverse reactions such as hepatotoxicity. In addition, research on paraptosis-associated ICD markers has also garnered attention. Previous studies have shown that paraptosis can be activated by controlling cell swelling and vacuolization through the large-conductance potassium big potassium (BK) channel, accompanied by the expression of ICD markers [164].

Based on this phenomenon, researchers have found that extending the activation of the BK channel leads to the overexpression of Hsp60, Hsp70, Hsp90, and gp90, releasing high mobility group B-1 (HMGB1), and enhancing the immunogenicity of rat glioma T9 cells4. However, the latest research has found that the release of HMGB1 is not significant and ICD markers are atypical in LD90 dose of verteporfin-based PDT, questioning the current mainstream practice of using HMGB1 as a marker for PDT-induced paraptosis [163].

In summary, the consequences and determining factors of paraptosis are still not fully understood and require further research. However, paraptosis has the potential to be an effective approach for killing tumor cells and activating the immune system. By further combining paraptosis and PDT in future research, the application potential of paraptosis in PDT for cancer treatment can be more fully explored, making it a more effective anti-tumor strategy.

## Other

### Application of PDT in PARP-1-dependent cell death

**Fig. 3:**
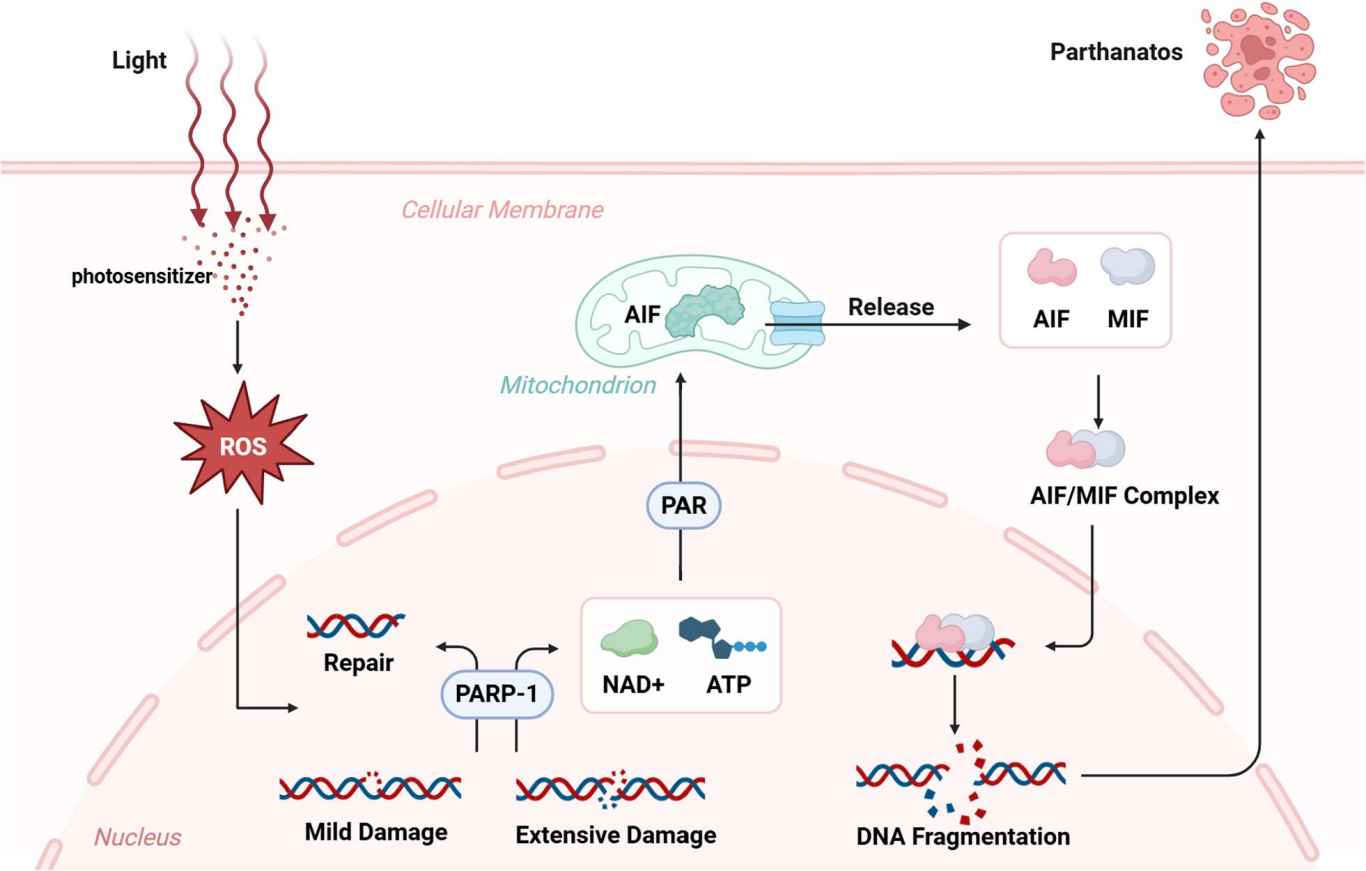
Schematic diagram of the process by which PARP-1-dependent cell death occurs when PDT is applied. The diagram illustrates the anti-tumor mechanism of PDT through non-apoptotic cell death pathways, specifically parthanatos, a PARP1-dependent and caspase-independent form of regulated cell death. PDT induces the generation of ROS, leading to DNA damage and the activation of PARP-1. PARP-1 is involved in repairing mild DNA damage through NAD + and ATP consumption. Extensive damage to DNA causes overactivation of PARP1, upregulation of poly(ADP-ribose) (PAR) synthesis, and depletion of NAD⁺ and ATP. This triggers the release of AIF from mitochondria. AIF interacts with MIF, forming an AIF/MIF complex that promotes DNA fragmentation in the nucleus. This process highlights a non-apoptotic cell death mechanism, leading to tumor destruction

Parthanatos is a type of programmed cell death dependent on poly(ADP-ribose)-polymerase 1 (PARP-1), a member of PARP enzyme family that also includes PARP1, PARP2, and tankyrases. Parthanatos is characterized as a PARP1-dependent and caspase-independent regulated cell death pathway. Studies have generally concluded that the activation of PARP-1 is the most classical pathway for inducing parthanatos among the PARP enzyme family members activated upon DNA damage. The response of PARP-1 varies greatly depending on the extent of DNA damage. Under mild DNA damage, PARP-1 and some proteins near the damage site can accelerate the DNA repair process by promoting the recruitment of DNA repair effector proteins [165]. However, when DNA damage is severe, PARP-1 is over-activated, resulting in the accumulation of poly(ADP-ribose) (PAR) near the damage site. Since NAD+ is a direct substrate for the synthesis of PAR, this process leads to drastic depletion of NAD+  [166]. NAD+ also serves as a cofactor in many redox reactions, such as the tricarboxylic acid cycle, glycolysis, and the pentose phosphate pathway [167], suggesting that the concomitant presence of parthanatos may affect the body’s oxygen utilization, further helping to promote cell death. In addition, PAR translocates from the nucleus to the mitochondria due to the massive depletion of NAD+  [168], triggering the release of apoptosis-inducing factor (AIF) from the mitochondria. The precise mechanism of AIF release remains incompletely elucidated. Proposed pathways indicate that NAD⁺ depletion induces mitochondrial membrane depolarization and AIF conformational changes. Additionally, PAR polymers directly interact with mitochondrial AIF, facilitating its release [169]. Released AIF binds to macrophage migration inhibitory factor (MIF) to form the AIF/MIF complex in the cytoplasm. Immediately after that, the AIF/MIF complex will re-translocate into the nucleus, leading to chromatin condensation and DNA fragmentation, which ultimately results in the end of cell death [170–172], and may have a facilitating effect on other modes of death, such as autophagy, ferroptosis, etc. (Fig. 3). Huang et al. [173] summarized the biochemical characteristics of the cells at the onset of parthanatos, with genetic features that suggested that PARP-1 can be used as a therapeutic target for some diseases, such as stroke, trauma, and diabetes models [174]. Here, we focus on the role of PARP-1 in PDT against tumors.

In PDT therapy, the combination of photosensitizer, light, and oxygen can trigger photochemical processes leading to the formation of ROS, which interact with cellular structures and lead to selective destruction of irradiated tissues [42]. It has been suggested that parthanatos during PDT may be initiated by nuclear-localized ROS generation, resulting in DNA damage and subsequent PARP overactivation. While ROS have been shown to elicit parthanatos [166], only a few studies have described the regulatory processes in response to PDT. Soriano et al. [175] investigated the cell death mechanisms induced by two photosensitizers in a paired model of non-tumorigenic (MCF-10A) and tumorigenic (SKBR-3) human breast cell lines. They found that the non-tumorigenic MCF-10A cells predominantly underwent PARP-dependent cell death, accompanied by AIF translocation. This process can be significantly inhibited by PARP inhibitors, thus increased the viability of MCF-10A cells. The study provides first evidence for the induction of parthanatos by PDT. From a translational perspective, adjunctive use of PARP inhibitors may confer a selective cytoprotective effect on normal tissues during PDT. In addition, the experimental results also showed that at lower concentrations of photosensitizers the resistance of non-tumorigenic cell lines was significantly higher than that of tumorigenic cell lines, confirming that PDT is less effective in healthy tissues and has certain advantages over traditional treatment modalities.

### PDT in lysosomal-dependent cell death

Lysosome-dependent cell death (LDCD) was first proposed by Christian de Duve [176], who suggested that the modality is capable of further inducing cellular and tissue autolysis through the osmotic delivery of hydrolytic enzymes to the cytoplasm via the cell membrane after their accommodation. Lysosomal membrane permeabilization (LMP) and the subsequent release of lysosomal protein hydrolases, including tissue proteases, are able to induce lysosomal cell death, leading to mitochondrial dysfunction and apoptosis [177]. Many cell death pathways ultimately lead to LMP [178]. However, in LDCD, LMP is the primary cause of death, not merely a downstream consequence. Kurz et al. [179] found that ROS can trigger LMP necessary for cell death, such as oxidative stress with some of the disease-induced LMPs. Under oxidative stress, excess H_2_O_2_ undergoes a redox-active iron reaction in lysosomes, generating highly reactive hydroxyl radicals, which further lead to lipid peroxidation and disruption of lysosomal membrane proteins, ultimately making the lysosomal membrane less stable. These changes suggest a possible correlation with the ROS changes that often accompany PDT.

Dos Santos et al. [85] have found that PDT using methylene blue as a photosensitizer (MB-PDT) can kill metastatic human breast cancer in vitro, and that LMP can be induced by MB-PDT. This conclusion is derived from their observation of increased cytoplasmic cathepsin B activity following MB-PDT. The key point is that the role of LMP extends beyond triggering general cell death; it acts as a critical upstream regulatory node that determines the specific death pathways activated in a cell-type-dependent manner. This mechanistic insight explains the therapy's selectivity. For example, inhibiting cathepsin B with CA-074 not only reduces cytotoxicity but also suppresses the key necroptosis marker pMLKL. This finding reveals a direct crosstalk between LDCD and necroptosis in malignant cells. Interestingly, LMP occurs in both normal and cancer cells, indicating that the selectivity of MB-PDT does not stem from LMP itself but from the differential ability of cells to respond to LMP. In tumor cells with defective antioxidant defenses, abundant labile iron, and high PUFAs, LMP initiates a lethal cascade involving necroptosis and ferroptosis. In contrast, non-tumor cells, with their robust antioxidant capacity, can buffer this oxidative stress, thereby preventing the activation of these lethal pathways downstream of LMP.

### Application of PDT in immunogenic cell death

In immunotherapy against tumors, including but not limited to the use of immune checkpoint inhibitors (ICIs), lymphocyte-activated cytokines, chimeric antigen receptor CAR-T cells, etc., but due to the easy off-target distribution of ICIs and the inhibitory tumor microenvironment of ICI-hypo-responsive patients [180], the modulation of the tumor immune microenvironment is of particular importance in immunotherapy, and in which ICD is very promising.

Researchers have found that ICD is capable of regulating tumor immunity through the release of antigenic and danger-associated molecular patterns (DAMPs), including tumor cell surface calreticulin (CRT), as well as some other factors [181], in which Panaretakis et al. [182] and Michaud et al. [183] demonstrated that, under conditions of endoplasmic reticulum stress, CRT is able to be translocated to the tumor cell surface and bind to its surface CD91 receptor to promote the recognition of tumor-associated antigens released by tumor cells by antigen-presenting cells, thereby activating tumor cell autophagy. In addition, the released tumor-associated antigens can recruit DCs, macrophages, and monocytes [183, 184], which continue to be delivered to T cells after phagocytosis of antigens, enhancing the immune response conversely. Notably, Kroemer et al. [185] also found that DAMP was shown to promote the maturation of DCs while DCs and others were delivered to T cells, suggesting a dual role of DAMP on DCs in the ICD model and a correlation with the intrinsic immune response and the induction of adaptive immune responses.

The treatment of PDT associated with ICD is relatively complete, and Showalter et al. [186] summarized three types of ICD induction, namely type I ICD inducers as chemotherapeutic agents, type II ICD inducers relying on physical methods, and other ICD inducers. In Hypericin-based PDT (HYP-PDT), Garg et al. [187] found that HYP-PDT resulted in a rise in phagocytosis of tumor cells by Mf4/4 phagocytes and T24 cells exposed to such PDT were observed to release DAMP in addition to a high level of NO, whereas the level of IL-10 was reduced to the lowest level, suggesting the presence of an active immune response. Another in vitro study by Grag et al. [188] found that if T24cells were pretreated with L-histidine, an oxygen quencher that inhibits ROS-dependent effects, it resulted in the loss of CRT exposure as well as ATP secretion in the cells, signifying that ROS play a role. While the direct application of L-histidine as a quenching agent is technically straightforward in cell culture but challenging in vivo due to systemic delivery and specificity issues, this study provides fundamental molecular insight. Dewaele et al. [26] found that autophagy prevents HYP-PDT-induced death, while ATP secretion is not required to induce autophagy. Researchers initiated a study on the role of autophagy in ICD, and the results showed that HYP-PDT treatment leads to the accumulation of oxidative damage proteins instead of being removed during autophagy, which results in an enhanced ICD response [186]. In addition, the results also showed that autophagy inhibition increased the maturation of DCs co-cultured with HYP-PDT-treated tumor cells, and in summary, this PDT therapy was able to lead to enhanced ICD as well as a significant maturation of DCs. Panzarini et al. [189] performed a similar study using another photosensitizing drug, Rose Bengal acetate (RBAc), and similarly found that the tumor cell ROS generation in tumor cells and ultimately led to autophagy and apoptotic cell death in HeLa cells.

### Application of PDT in granzyme-mediated cell death

Evangelou et al. [190] found in superficial/nodular basal cell carcinoma and intraepidermal squamous cell carcinoma (Bowen’s disease) that intra-lesional PDT treatment was able to induce apoptosis through the non-dependent apoptotic mediators involved in the intrinsic apoptotic pathway (including cystathionase 3 as well as granzyme B) and pro-apoptotic markers BAX and BAK expression in the pathways to induce apoptosis in tumor cells of basal cell carcinoma and Bowen disease. In addition, Valančiūtė et al. [191] also found that in MB-PDT/low-energy LED phototherapy, on the one hand, it was able to lead to a reduction in the proliferation of non-small-cell lung cancer (NSCLC) cell lines, and the researchers found that the therapy induced necrosis associated with a decrease in the expression of the anti-apoptotic protein Bcl2, and detected ICD markers in the NSCLC cells, CRT and major histocompatibility complex-1 (MHC-1) expression were elevated, suggesting that PDT promotes an immune response. On the other hand, this study also identified a role for the granzyme B pathway in tumor cell apoptosis, and the researchers found that a rise in granzyme B activity led to enhanced activated CD8+ T cell-mediated cell-killing lysis of lung cancer cells. In summary, granzyme B plays a dominant role in granzyme-mediated cell death and its induction of the intrinsic apoptotic pathway is similar to that of ICD above.

## Conclusion

PDT represents an emerging modality in oncology that directly kills tumor cells or disrupts the tumor microenvironment by activating photosensitizers in vivo to generate ROS. Previous studies have generally considered apoptosis to be the primary mode of PDT-induced cell death, but accumulating evidence indicates that non-apoptotic pathways-such as necrosis, ferroptosis, pyroptosis, mitotic catastrophe, and paraptosis collectively overcome apoptosis resistance and improve therapeutic efficacy. Additionally, while primarily cytoprotective, autophagy-related pathways also contribute to cell death under certain PDT conditions. To explore these non-apoptotic cell death mechanisms and their applications in tumor therapy, this review systematically analyses their molecular mechanisms and synergistic effects with PDT. Autophagy has a dual role, necrosis can act synergistically with immune activation, ferroptosis, pyroptosis, mitotic catastrophe, and paraptosis immune activation, ferroptosis amplifies ROS and oxygen production, pyroptosis enhances immunogenicity and combats immunologically “cold” tumors, mitotic catastrophe and paraptosis exploit genomic instability and organelle stress. By harnessing these death pathways, PDT effectively overcomes resistance in tumors to conventional therapies, demonstrating its potential for widespread clinical application. Future studies should focus on optimizing photosensitizers and light conditions in combination with other therapeutic means to enhance the clinical efficacy of PDT.

## Data Availability

No datasets were generated or analysed during the current study.

## Abbreviations

AIF: Apoptosis-inducing factor
AIE PSs: Photosensitizers with aggregation-induced emission characteristics
AIEgen: Aggregation-induced emission luminogen
5-ALA: 5-Aminolevulinic acid
AMD: Age-related macular degeneration
Ce6: Chlorin e6
CRT: Calreticulin
DAC: Decitabine
DC: Dendritic cell
FIH: Factor inhibiting HIF1
GPX4: Glutathione peroxidase 4
GSDMD: Gasdermin-D
GSDME: Gasdermin-E
HIF1: Hypoxia-inducible factor 1
HMOX1: Heme oxygenase-1
HYP-PDT: Hypericin-based PDT
ICB: Immune checkpoint blockade
ICD: Immunogenic cell death
ICIs: Immune checkpoint inhibitors
IFN-γ: Interferon-γ
JNK: C-Jun N-terminal kinase
LDCD: Lysosome-dependent cell death
LDH: Lactate dehydrogenase
LED: Light-emitting diode
LMP: Lysosomal membrane permeabilization
MAPK: Mitogen-activated protein kinase
MB-PDT: PDT using methylene blue as a photosensitizer
MHC-1: Major histocompatibility complex-1
MIF: Migration inhibitory factor
N-GSDMD: N-terminal gasdermin-D
NFκB: Nuclear transcription factor-κB
NSCLC: Non-small-cell lung cancer
PAR: Poly(ADP-ribose)
PARP-1: Poly(ADP-ribose)-polymerase 1
Pc13: Zinc (II) phthalocyanine photosensitizers
PDT: Photodynamic therapy
PHD: Prolyl hydroxylase domain protein
PpIX: Porphyrin IX
RB: Rose Bengal
RBAc: Rose Bengal acetate
ROS: Reactive oxygen species
TBD: 1,1,2,2-Tetraphenylethene-benzolc[1,2,5]thiadiazole-2-(diphenyl methylene) malononitrile
TDC: Chlorophyll derivative 5,10,15,20-tetrakis (3,4-dihydroxyphenyl) chlorin
TPSS: Two-photon photosensitizer
WST11 VTP: WST11-loaded vascular-targeted photodynamic therapy
ZnPc: Zinc phthalocyanin
